# Synthesis and Spectroscopic Characterization of Water-Soluble Fluorescent Ag Nanoclusters

**DOI:** 10.1155/2013/261648

**Published:** 2013-05-20

**Authors:** Chengzhi Zheng, Huiping Wang, Lingzhi Liu, Manjun Zhang, Jiangong Liang, Heyou Han

**Affiliations:** State Key Laboratory of Agricultural Microbiology, College of Science, Institute of Chemical Biology, Huazhong Agricultural University, Wuhan 430070, China

## Abstract

Water-soluble fluorescent Ag nanoclusters (NCs) were synthesized at room temperature with sodium dodecyl sulfonate (SDS) as a protective agent. The effects of synthetic conditions on the fluorescence properties of Ag NCs were investigated. The results show that the fluorescence intensity of Ag NCs strongly depends on the synthetic conditions, such as the molar ratio of AgNO_3_ versus SDS and sodium borohydride (NaBH_4_), the reaction time, and the pH value of the reaction solution. Under the optimum conditions, the as-prepared Ag NCs exist in face-centered-cubic phase with an average size of 2 nm. Fluorescence spectra of Ag NCs show emission peaks at 365 nm for different excitation wavelength. Resonant absorptions are observed at 203 nm and 277 nm in the absorption spectrum, which can be used to establish the electronic levels in the Ag NCs system.

## 1. Introduction

In recent years, noble metal nanoclusters have received considerable attention due to their marvelous optical, physical, and electrical properties for use in sensing, biological imaging, and single-molecular spectroscopy [1–7]. Among the various noble metal clusters reported to date, low-nuclearity Ag NCs have been the subject of intense interest due to their low toxicity, ultrasmall size (<2 nm), and good fluorescence properties [8, 9]. In the past years, several synthetic strategies have been proposed for the preparation of Ag NCs such as radiolytic [10, 11], photochemical [12], sonochemical [13], and chemical reduction approaches in the presence of various scaffolds, including polyelectrolyte [11], dendrimers [14], peptides [15], and DNA [16]. However, these methods usually involve complex processes or require expensive raw materials.

In the present work, we reported a facile one-pot synthetic route to prepare water-soluble fluorescent Ag NCs with the help of SDS. Compared with the previous methods using DNA, polymers, or peptides as templates, SDS, as a common commercial surfactant, is much simpler and more economical.

## 2. Experimental Section

### 2.1. Synthesis of Ag NCs

In a typical experiment, a freshly prepared mixture solution of AgNO_3_ (1.0 mM) and SDS (50 *μ*M) was incubated in the ice bath for *ca*. 40 min and then reduced by the addition of NaBH_4_ (2.0 mM) with vigorous stirring for another 3 h at room temperature. The pH value of the reaction solution was adjusted by acetate buffer solution.

### 2.2. Characterization

The UV-Vis absorption spectrum was obtained in the range of 190–700 nm, with 1.0 cm × 1.0 cm quartz cuvette on a Thermo Nicolet Corp model Evolution 300 (America). All fluorescence spectra were recorded by a Shimadzu RF-5301PC Spectrofluorometer (Japan) equipped with a 20 kW xenon discharge lamp as a light source. The size and morphology of Ag NCs were acquired using a FEI Tecnai G20 transmission electron microscope (TEM) with an acceleration voltage of 200 kV. Powder X-ray diffraction (XRD) was recorded using a D/Max-3B X-ray diffractometer (Rigaku International Corp., Japan) with Cu K_*α*_ source (40 kV and 30 mA). All pH measurements were tested with a Model MP120 pH meter (Mettler-Toledo Instruments Ltd., Swiss).

## 3. Results and Discussion

**Figure 1:**
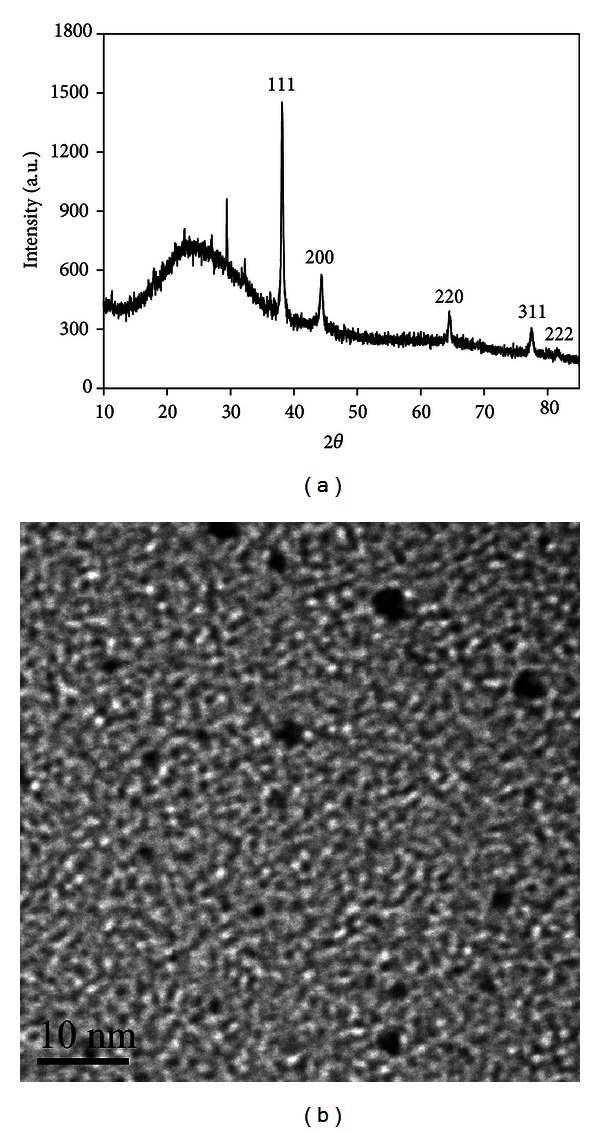
(a) The XRD pattern and (b) TEM image of Ag NCs.

A typical XRD pattern of Ag NCs is shown in Figure 1(a). It is seen that the major peaks locate at 2*θ* values of 38.12°, 44.28°, 64.43°, 77.48°, and 81.54°, which correspond to (111), (200), (220), (311), and (222) planes, respectively [17]. The peaks position and relative intensity are in good agreement with the values of Ag in the standard card (JCPDS number 04-783) and reveal that Ag NCs are in face-centred-cubic structure. The corresponding TEM image (Figure 1(b)) indicates that the average size of Ag NCs is about 2 nm and the nanoclusters are spherical in shape.

**Figure 2:**
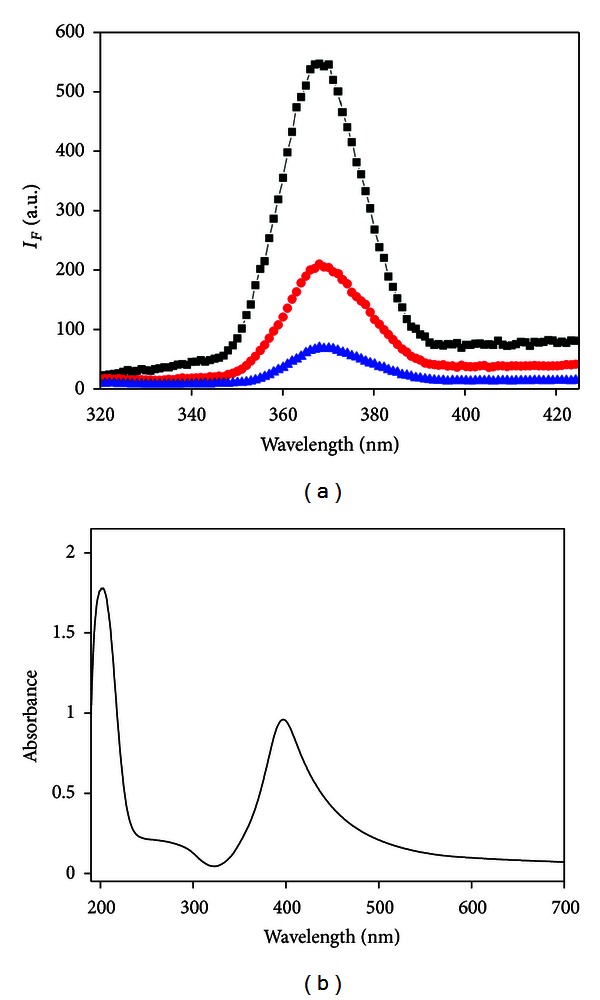
(a) Fluorescence emission spectra of Ag NCs at *λ*_ex_ = 220 nm (black curve), 240 nm (red curve), and 260 nm (blue curve), respectively; (b) UV-visible absorption spectrum of Ag NCs.

**Scheme 1:**
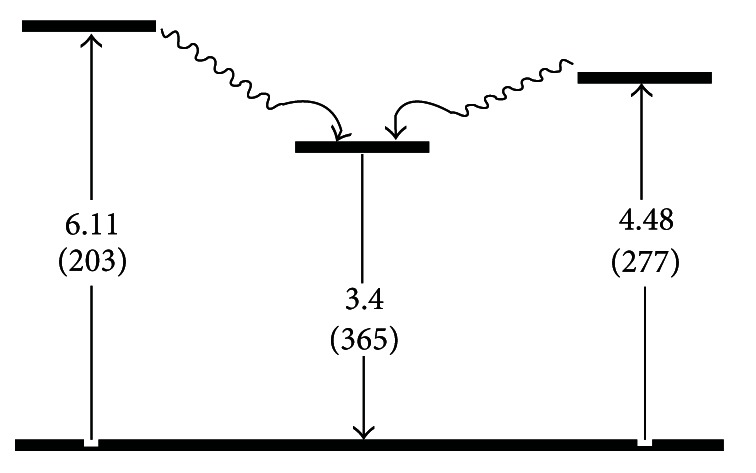
Schematic diagram of energy levels explaining the fluorescence emission spectrum observed for Ag NCs. Energy values are given in eV (and nm inside parenthesis).

Figure 2(a) manifests the fluorescence spectra of Ag NCs. Compared with the reported fluorescence spectra of Ag NCs that contain several emission peaks under different excitation wavelengths [18], the maximal emission of our Ag NCs is only observed at 365 nm with excitation wavelength (*λ*_ex_) in the range of 220–260 nm. Assuming that one cluster has only one main specific emission peak [19], we can infer that there is only one cluster in our synthesized Ag NCs. The quantum yield (QY) of Ag NCs was estimated by using quinoline sulfate as a reference sample [15, 20]. The QY of as-prepared Ag NCs is 0.84%, which is comparable with the QY of Ag NCs reported in the reference [21, 22].  Figure 2(b) shows the UV-Vis spectrum of Ag NCs. An apparent peak at 397 nm was observed in the spectrum, and it can be assigned to surface plasmons resonance arising from collective oscillations of the valence electrons in the electromagnetic field of the incident light. There are two absorption peaks at 203 nm and 277 nm, respectively, which are consistent with the result reported by Siwach and coworkers [23]. The two absorption peaks can be ascribed to resonant peaks. Fluorescence from the noble metal has been well studied and mainly assigned to transitions of the electrons between the conduction band below the Fermi level and holes in the d bands [24]. A detailed study of fluorescence properties of the metal nanoclusters [25] allows us to ascribe this to transitions involving more than one excited state. The fluorescent behavior of Ag NCs observed is summarized in Scheme 1. The peaks at 277 nm and 203 nm correspond to the transitions between the ground state, the first excited state, and the second excited state. The only way fluorescence emission at 365 nm from both these two absorption peaks can be achieved is through an internal conversion involving these excited states.

**Figure 3:**
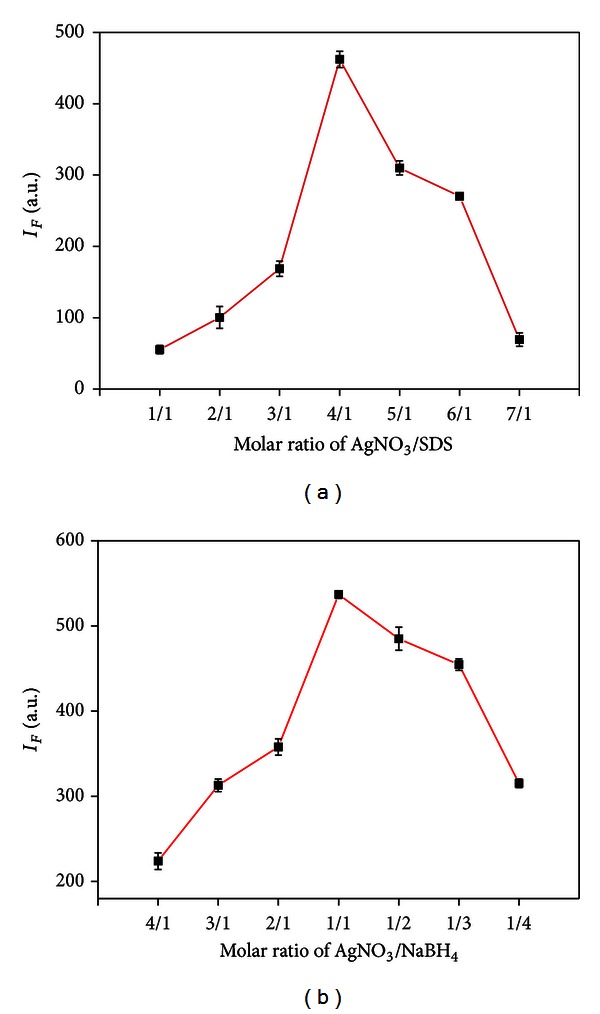
The effect of molar ratio of (a) AgNO_3_/SDS and (b) AgNO_3_/NaBH_4_ on the fluorescence intensity of Ag NCs.

The effects of synthetic conditions on the fluorescence properties of Ag NCs are very important and should be optimized carefully. Firstly, the influence of the molar ratio of AgNO_3_ versus SDS and NaBH_4_ on the fluorescence intensity of Ag NCs was studied.  Figure 3(a) shows the effects of molar ratio of AgNO_3_ versus SDS on fluorescence intensity of Ag NCs at 365 nm wavelength. And the maximum intensity was acquired when the molar ratio was 4 : 1. From Figure 3(b), we can also find that when the molar ratio of AgNO_3_ versus NaBH_4_ was 1 : 1, Ag NCs adopt the maximum fluorescence intensity.

**Figure 4:**
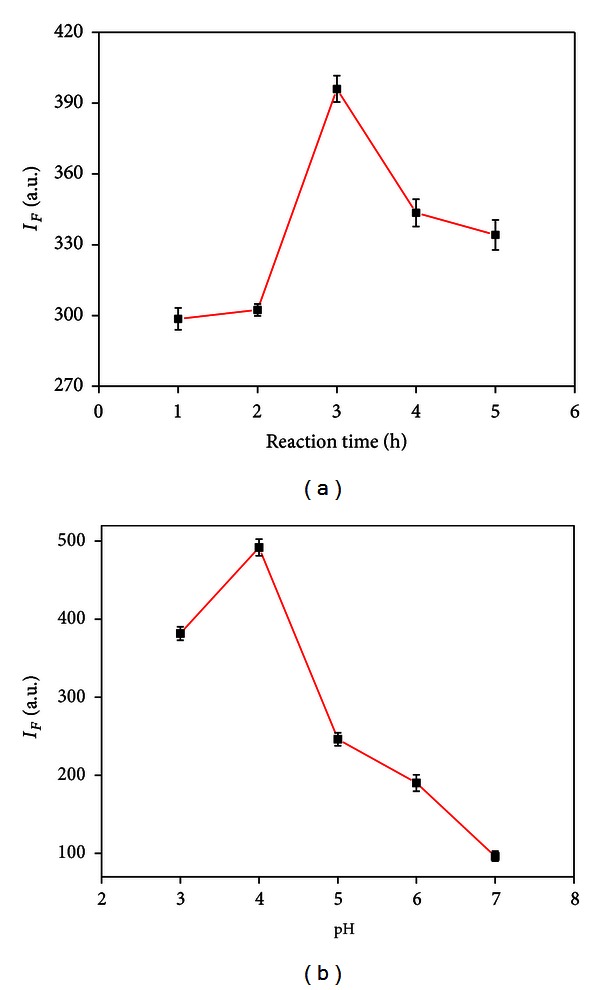
The effect of (a) reaction time and (b) pH value on the fluorescence intensity of Ag NCs.

We also investigated the effect of the reaction time and pH values on the fluorescence intensity of as-prepared Ag NCs. As shown in Figure 4(a), the optimum reaction time is 3 h.  Figure 4(b) indicates the effects of pH values on the fluorescence intensity. The maximum intensity was obtained at pH 4.0, and the nanocluster synthesized at either higher or lower pH value showed weaker fluorescence intensity. At higher pH, the hydroxide ions will compete with sulfonate ions for the complexing of silver ions and even formed silver oxide. At lower pH, the existence of hydrogen bond will weaken the interaction between sulfonic acid groups and silver atoms. In our experiment, the optimum pH value that balances the influence of both factors in the SDS-assisted synthesis of Ag NCs was found to be 4.0.

The synthetic process of Ag NCs mainly involves two steps. Firstly, Ag^+^ was reduced to Ag sol by NaBH_4_ and immediately was coordinated with borohydride ions. Then, sulfonic acid groups substituted the borohydride ions and then formed Ag NCs, since they have a better affinity for metal atoms. We expect SDS to be an ideal template for at least three reasons: (1) SDS carries sulfonic acid groups capable of coordinating with Ag^+^; (2) SDS exhibits necessary hydrophobicity due to the presence of dodecyl groups, and previous studies found that hydrophobic regions facilitated the formation of metal nanoclusters [1]; (3) most importantly, SDS as protective agent is much more simpler and economical.

## 4. Conclusion

A convenient chemical reduction method for the synthesis of water-soluble fluorescent Ag NCs has been successfully developed. The ease of synthesis as well as the utilization of SDS makes these inexpensive and fluorescent Ag NCs attractive due to their good reproducibility and great potential for large-scale synthesis. Furthermore, the stability and excellent fluorescent properties of these Ag NCs enable them to find widespread applications in bioimaging, chemical, and biosensing.
